# Lack of replication of genetic predictors for the rheumatoid arthritis response to anti-TNF treatments: a prospective case-only study

**DOI:** 10.1186/ar2990

**Published:** 2010-04-27

**Authors:** Marian Suarez-Gestal, Eva Perez-Pampin, Manuel Calaza, Juan J Gomez-Reino, Antonio Gonzalez

**Affiliations:** 1 Laboratorio Investigacion 10 and Rheumatology Unit, Instituto de Investigacion Sanitaria-Hospital Clinico Universitario de Santiago, Travesia Choupana sn., Santiago de Compostela, 15706, Spain; 2 Department of Medicine, University of Santiago de Compostela, San Francisco sn., Santiago de Compostela, 15782, Spain

## Abstract

**Introduction:**

We aimed to replicate the strong associations that a recent genome wide association study (GWAS) has found between 16 single nucleotide polymorphisms (SNPs) and response to anti-tumour necrosis factor (TNF) treatment in 89 patients with rheumatoid arthritis (RA). This study is very important because, according to published simulations, associations as strong as the reported ones will mean that these SNPs could be used as predictors of response at the individual level.

**Methods:**

Disease activity score (DAS28) was evaluated in 151 anti-TNF treated patients with RA of Spanish ancestry at baseline and every 3 months thereafter. Genotypes of the 16 putative predictor SNPs were obtained by single-base extension. Association between the relative change in DAS28 and SNP genotypes was tested by linear regression. In addition, logistic regression was applied to compare genotypes in non-responders (n = 34) versus good-responders (n = 61) following the EULAR response criteria.

**Results:**

None of the analyses showed any significant association between the 16 SNPs and response to anti-TNF treatments at 3 or 6 months. Results were also negative when only patients treated with infliximab (66.9% of the total) were separately analyzed. These negative results were obtained in spite of a very good statistical power to replicate the reported strong associations.

**Conclusions:**

We still do not have any sound evidence of genetic variants associated with RA response to anti-TNF treatments. In addition, the possibility we had envisaged of using the results of a recent GWAS for prediction in individual patients should be dismissed.

## Introduction

Anti-tumor necrosis factor (anti-TNF) therapies have revolutionized the treatment of rheumatoid arthritis (RA) [1,2]. Three drugs of this type, infliximab, etanercept, and adalimumab, have been used with success in hundreds of thousands patients with RA around the world. New drugs targeting TNF are in development or have been recently approved [3]. The beneficial effects of these drugs include a better quality of life; control of inflammation, stiffness, and pain; and slowing progression to joint erosions and deformity. It seems also that they are able to decrease cardiovascular risk and overall mortality of patients with RA [4,5]. However, there is a significant percentage of patients who do not obtain these advantageous effects [1-3]. In some of these patients, this lack of response is primary, from the start of the treatment, whereas others develop resistance to treatment after a period of initial response. Unfortunately, there are no useful predictors to forecast what the clinical response of a specific patient will be. This has led to an unsatisfactory trial-and-error approach in the selection of drugs, meaning that some patients will miss an effective treatment at a critical window of opportunity [6] and that health service resources will be wasted. In response to this challenge, multiple lines of research are looking for predictors of response to anti-TNF therapies among patient clinical features, synovial tissue biomarkers, blood proteins, or genetic variants [7-10]. Very promising, though preliminary, findings have been reported in this last field. Sixteen single-nucleotide polymorphisms (SNPs) with an important association with response to treatment were identified in a recent genome-wide association study (GWAS) [7]. In our view, the most remarkable aspect of these findings was the marked effect size of each SNP, with levels very rarely found in genetic studies of complex traits. All showed an odds ratio (OR) of more than 3.5 in the comparison between patients with good response and non-responders. Some of these SNPs showed effect sizes of an OR of more than 10. If confirmed, these effects, together with minor allele frequencies of more than 12%, will allow the prediction of response to anti-TNF treatments with great accuracy at the level of the individual patient [11]. The limitation of this study was that only 89 patients were included, and even very significant results in a study of this size are uncertain. Our objective has been to provide the necessary replication to these exciting findings with the expectation that at least a few of them will be confirmed. This will be a first step before proceeding to prospective clinical studies to assess their utility in clinical practice.

## Materials and methods

### Patients

A group of 151 patients with RA were followed prospectively at the Rheumatology Unit of the Hospital Clinico Universitario de Santiago to study the efficacy of anti-TNF therapy. All of them were of European (Spanish) ancestry. Only patients who were naïve with respect to biologic treatments were included. Patients were systematically evaluated at the initiation of therapy and every 3 months thereafter. Evaluations included painful and swollen joint counts, visual analog scales of pain, global health assessments by the patient and the physician, erythrocyte sedimentation rate (ESR), C-reactive protein (CRP), health assessment questionnaire (HAQ), and disease activity score using 28 joint counts (DAS28). Clinical characteristics are detailed in Table 1. All participants gave their informed consent for inclusion, and the study and procedures were approved by the clinical research ethics committee of Galicia.

**Table 1:** Clinical characteristics of the patients in this study and of those in the report by Liu and colleagues [7]

Variable	This study	**Data from **[7]	*P *value
Age in years, mean ± SD	54.2 ± 13.1	57 ± 13.5	0.06
Age in years at diagnosis, mean ± SD	46.2 ± 13.5	47 ± 15	0.3
Women, percentage	84.8	75	0.06
Disease duration in years, mean ± SD	7.8 ± 6.6	8 ± 8	0.4
Current smokers, percentage^a^	9.1	15	0.3
Ever smokers, percentage^a^	25.0		
			
Rheumatoid factor-positive, percentage	68.9	83.8	0.01
Anti-CCP-positive, percentage^a^	86.3	61.9	0.0001
Shared epitope-positive, percentage^a^	58.3		
Antinuclear antibody-positive, percentage^a^	29.8		
			
HAQ at baseline, mean ± SD	1.4 ± 0.7	1.1 ± 0.6	0.0005
DAS28 at baseline, mean ± SD	5.6 ± 1.2	5.2 ± 0.8	0.002
DAS28 ≥ 5.1, percentage	70.2		
3.2 ≤ DAS28 > 5.1, percentage	26.5		
DAS28 at 12 to 16 weeks, mean ± SD	3.6 ± 1.4	3.7 ± 1.3	0.2
DAS28 at 6 months, mean ± SD	3.5 ± 1.4		
Good responders at 12 to 16 weeks, percentage	40.4	34.8	0.6
Non-responders at 12 to 16 weeks, percentage	22.5	25.8	0.6
Good responders at 6 months, percentage	43.7		
Non-responders at 6 months, percentage	21.8		
			
Anti-tumor necrosis factor drug			
Infliximab, number	101	32	0.01
Etanercept, number	35	39	0.02
Adalimumab, number	15	18	0.05

^a^Data were available for 102 patients for anti-cyclic citrullinated peptide (CCP) antibodies, antinuclear antibody, and shared epitope and for 88 patients for smoking habits. DAS28, disease activity score using 28 joint counts; HAQ, health assessment questionnaire; SD, standard deviation.

### Assessment of the efficacy of the treatment

We used the same procedures described in Liu and colleagues [7] to make our results comparable. Response to anti-TNF treatments was assessed with the DAS28 [12]. The primary outcome was the quantitative variable relDAS28, which is the relative change in DAS28 between baseline and the time of evaluation. Presented as a percentage, this variable is calculated as follows:

A secondary outcome was the European League Against Rheumatism (EULAR) response classification in good, moderate, or non-responders [13]. Good responders have ΔDAS28 of at least 1.2 and DAS28 at 3 months of not more than 3.2; moderate responders have (a) ΔDAS28 of at least 1.2 and DAS28 at 3 months of greater than 3.2 or (b) 0.6 < ΔDAS28 ≤ 1.2 and DAS28 at 3 months of not more than 5.1; and non-responders are those who do not fit into any of these categories.

### Genotypes

A total of 16 SNPs from Liu and colleagues [7] were analyzed (Table 2). Genotypes were obtained by single-base extension with the SNaPshot Multiplex Kit (Applied Biosystems, Foster City, CA, USA) and specific primers and probes (available in Additional file 1). The genotype call rate was 99.79%, allele frequencies were in Hardy-Weinberg equilibrium, and concordant results for the 16 SNPs were obtained in the 21 samples that were genotyped twice.

**Table 2:** Relationship of relDAS28 and single-nucleotide polymorphism genotypes and comparison of allele frequencies between responders and non-responders

Single-nucleotide polymorphisms	*P *value of relDAS28	MAF of responders, percentage (n/N)	MAF of non-responders, percentage (n/N)	**OR**^a^**(95% CI)**	*P *value
rs983332	0.6	25.9 (30/116)	27.9 (19/68)	1.11 (0.6-2.0)	0.8
rs928655	0.1	18.9 (23/122)	28.0 (19/68)	0.60 (0.3-1.2)	0.1
rs13393173	0.8	23.8 (29/122)	20.6 (14/68)	0.83 (0.4-1.7)	0.6
rs437943	0.05	38.5 (47/122)	25.0 (17/68)	0.53 (0.3-1.0)	0.06
rs10945919	0.7	27.9 (34/122)	23.5 (16/68)	0.79 (0.4-1.7)	0.5
rs854547	0.3	39.3 (48/122)	36.8 (25/68)	1.12 (0.6-2.1)	0.7
rs854548	0.9	23.0 (28/122)	23.5 (16/68)	1.03 (0.5-2.0)	0.9
rs854555	0.6	35.2 (43/122)	38.2 (26/68)	1.14 (0.6-2.0)	0.7
rs868856	0.6	32.8 (40/122)	36.8 (25/68)	1.19 (0.6-2.0)	0.6
rs7046653	0.5	32.0 (39/122)	36.8 (25/68)	1.23 (0.7-2.5)	0.5
rs2814707	0.8	25.4 (31/122)	29.4 (20/68)	1.22 (0.6-2.5)	0.6
rs3849942	0.4	23.0 (28/122)	29.4 (20/68)	1.41 (0.7-2.5)	0.3
rs774359	0.2	26.2 (32/122)	36.8 (25/68)	1.64 (0.8-3.3)	0.1
rs6138150	0.5	14.0 (17/122)	14.7 (10/68)	0.94 (0.4-2.2)	0.9
rs6028945	0.9	12.3 (15/122)	13.2 (9/68)	1.09 (0.5-2.5)	0.9
rs6071980	0.9	18.9 (23/122)	16.2 (11/68)	0.83 (0.4-2.0)	0.6

^a^Odds ratios (ORs) were calculated as in Liu and colleagues [7], taking the allele associated with the non-responders in that report as the numerator of the odds, and the non-responder odds as the numerator of the OR. CI, confidence interval; MAF, minor allele frequency; n, number of minor alleles; N, total number of alleles; relDAS28, relative change in disease activity score using 28 joint counts between baseline and time of evaluation.

### Statistical analysis

Comparisons of the clinical characteristics of the RA patients included in the GWAS and in our study were done with the Student *t *test for data available as mean and standard deviation and with the chi-square test for contingency tables for frequency data. Analyses of the relationship between SNPs and treatment response were done as in Liu and colleagues [7] to make our results comparable in this aspect. Briefly, linear regression analysis between genotype data following a genetic additive model and relDAS28 as the continuous dependent variable was done. A *t *statistic was derived from the linear regression and used to calculate the *P *value of the association. This statistic is robust to deviations from normality of relDAS28. We also conducted logistic regression analysis between the groups of responders and non-responders. ORs and their 95% confidence intervals (CIs) were obtained using the non-responder group as the reference. This second analysis will be less powerful because the phenotype is transformed to a dichotomous variable and because the sample size is reduced by exclusion of the moderate responders. Statistical analyses were performed with a customized version of the Statistica 7.0 program (StatSoft, Inc., Tulsa, OK, USA). We visually explored the possibility that consideration of all of the SNPs jointly would discriminate between responder and non-responder patients. This analysis was done with the Co-Plot algorithm implemented in the Visual Co-Plot software [14,15]. Estimation of the statistical power for the linear regression analysis was done by transforming the reported *P *values and the number of samples in the corresponding correlation coefficients (*R*^2^). The values of *R*^2 ^and the number of samples in our study were used as input in the module for the F test in omnibus comparisons by linear regression of G*Power version 3.0.10 software [16].

## Results

**Figure 1:**
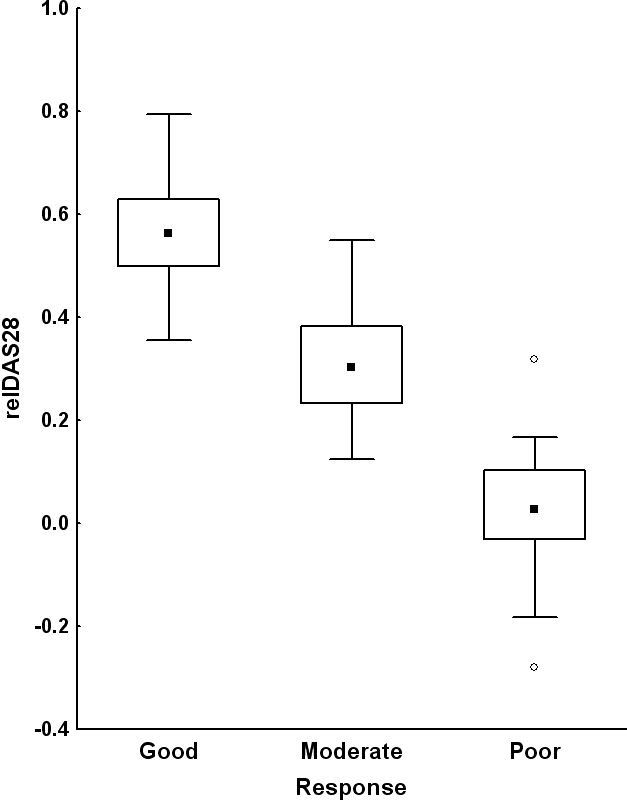
**Good correlation between outcome variables: EULAR (European League Against Rheumatism) response classification and relDAS28**. Medians, interquartile ranges, and non-outlier ranges are represented as dots, boxes, and whiskers, respectively. Empty dots represent outliers. relDAS28, relative change in disease activity score using 28 joint counts between baseline and time of evaluation.

The aim of our study was to replicate the strong association of 16 SNPs with response to anti-TNF therapy reported in a recent GWAS [7]. Therefore, we used the same variables and type of analysis. Data from the 151 patients with RA are shown in Table 1. Some of the characteristics of our study population were different from those of the patients analyzed in the GWAS [7]. Specifically, our patients showed a lower percentage of rheumatoid factor positivity, higher positivity for anti-CCP (anti-cyclic citrullinated peptide) antibodies, and higher baseline HAQ and DAS28 levels. There were 70.2% of patients with high disease activity at baseline as assessed by a DAS28 of greater than 5.1. In spite of this high activity, there were 40.4% and 43.7% of good responders at 3 and 6 months, respectively, and only 22.5% and 21.8% of non-responders at 3 and 6 months, respectively. The percentages of responders and non-responders were similar in the two studies. In contrast, the proportion of patients treated with each of the three anti-TNF drugs was different (Table 1). In our cohort, most patients were treated with infliximab (66.9%), followed by etanercept (23.2%) and adalimumab (9.9%). We also checked that there was a good correlation between the variable used as primary outcome in our analysis, relDAS28, and the EULAR response classification (Figure 1), allowing for consistency in the analyses.

**Figure 2:**
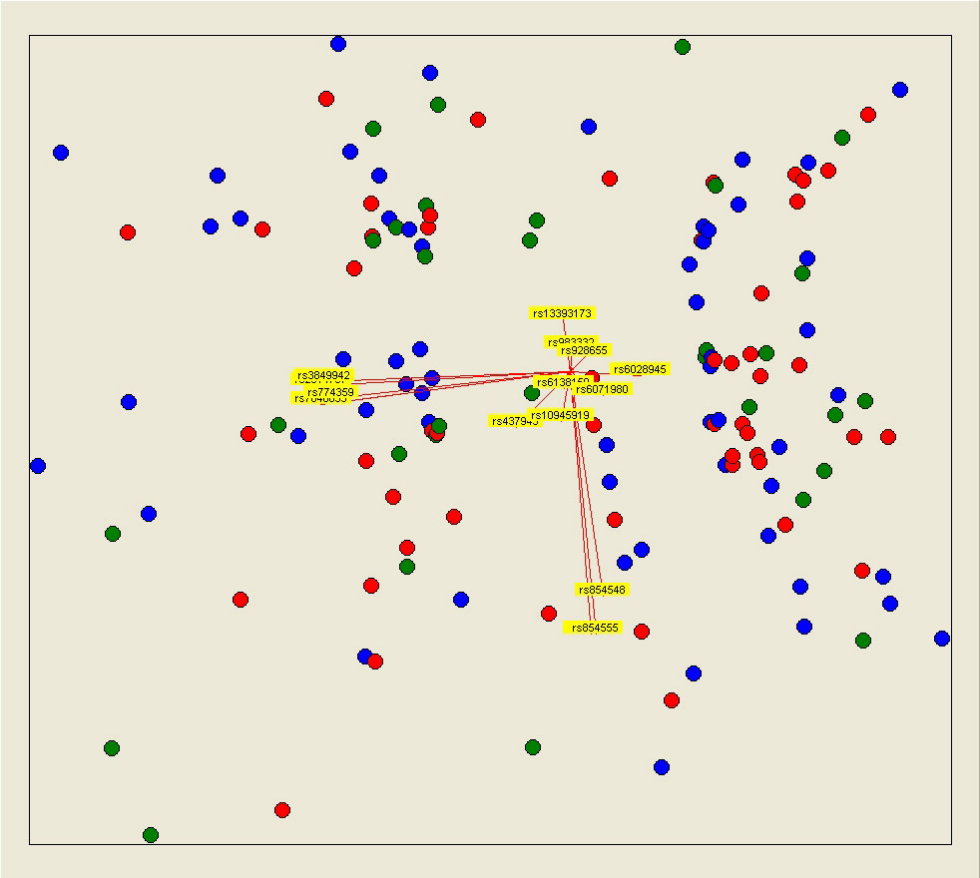
**Multivariate visual analysis showing that 16 single-nucleotide polymorphisms were not able to separate rheumatoid arthritis patients classified according to their EULAR (European League Against Rheumatism) response**. Responders are represented as blue dots, moderate responders as red dots, and non-responders as green dots. Yellow arrows represent the genotypes of each of the 16 single-nucleotide polymorphisms according to an additive model. This representation was obtained with Visual Co-Plot.

The relationship between the SNP genotypes and response to anti-TNF treatment at 3 months was evaluated by linear regression analysis between the genotypes and the continuous variable relDAS28. There was no association of any of the 16 SNPs with relDAS28 at 3 months (Table 2). Secondary analyses showed very similar results. Comparison of non-responders with good responders according to the EULAR criteria at 3 months did not show any significant association (Table 2). The most extreme OR (1.9, 95% CI 1.0 to 3.6) corresponded to rs437943 in the CNTDE1 locus but compared poorly with the previously reported OR (4.6, 95% CI 1.8 to 12.3). In addition, there was no association of relDAS28 with any of the 16 SNPs at 6 months or of the classification in responders and non-responders (Additional file 2). Finally, analysis of patients treated with infliximab, which represented 66.9% of our study, did not show any significant association between response and the SNPs (Additional file 2). Because of the small number of patients in the etanercept or adalimumab subgroups, no separate analyses of response to treatment were done. We also visually explored whether joint consideration of the 16 SNPs was able to discriminate between the different groups of patients according to their response to treatment, but patients with different responses did not show any clustering in identifiable groups in this analysis (Figure 2).

To interpret the above results, it was critical to assess whether our study had enough statistical power to replicate the previously reported associations. Power for the weakest association in the GWAS, which corresponds to rs928655 (*P *= 3 × 10^-5^), was larger than 95% for a *P *value of 0.002. It is important to remark that ORs from the GWAS are very likely heavily biased upwards as a consequence of the winner's curse affecting any GWAS and especially those of small size [17,18]. Therefore, this power estimate is valuable only in the context of the reported ORs taken at face value.

## Discussion

There is a great need of good predictors for RA response to the anti-TNF treatments [1-3,9]. The development and approval of new effective drugs for RA add to this urgency [3]. The recent GWAS from Liu and colleagues [7] was especially remarkable because it showed such strong associations that, according to published simulations [11], they could be used for prediction in individual patients. This is a characteristic that has not been found in any of the previous studies. However, the size of the study implied that results should be replicated before they could be taken at face value, as already acknowledged by the authors. We have tried to provide here the needed replication in the expectation that some of them will be confirmed and that validation in prospective studies will soon follow.

Unfortunately, in spite of the moderately larger sample size of our study and the corresponding very good power to detect this type of strong association, none of the associations was replicated. These results make it very unlikely that any of the 16 SNPs could have an association as strong as suggested by the previous GWAS [7]. It is possible that the differences between the patients with RA in the two studies could have had an effect on the lack of replication, but these differences were not large enough to completely explain the very divergent results. In addition, patients in the GWAS were predominantly of European ancestry as were all of the patients in our study. Therefore, it seems more likely that the original strong associations were due to random variation of allele frequencies in a study including more than 300,000 SNPs and to the heavy bias characteristic of GWASs of small sample size [17,18]. This possibility was already considered by us before beginning this study, but we judged that some SNPs would be replicated given that they showed low *P *values, five of them with *P *values of less than 10^-6 ^[7], and low *P *values are the best indication of the reproducibility of results [19].

## Conclusions

Our negative results imply that we still do not have any strong evidence supporting a significant role of genetic variation in the response to anti-TNF treatments. In addition, our results imply that none of the SNPs in our study will be useful as individual predictors of response to anti-TNF therapy, but do not exclude a weaker association.

## Abbreviations

anti-TNF: anti-tumor necrosis factor; CI: confidence interval; DAS28: disease activity score using 28 joint counts; EULAR: European League Against Rheumatism; GWAS: genome-wide association study; HAQ: health assessment questionnaire; OR: odds ratio; RA: rheumatoid arthritis; relDAS28: relative change in disease activity score using 28 joint counts between baseline and time of evaluation; SNP: single-nucleotide polymorphism; TNF: tumor necrosis factor.

## Competing interests

Roche Spain (Madrid, Spain) contributed to the funding of this project. However, the company had no input in the design of the study, the analysis, or the writing of the manuscript. The company did not have the right to early access to results or the right to interfere in any other way with the interpretation or reporting of the results. Therefore, the authors take exclusive and complete responsibility for the study.

## Authors' contributions

MS-G participated in the design of the study, genotyped the samples, and participated in the interpretation of the results and in writing the manuscript. EP-P participated in the acquisition of clinical data and collection of samples and in the analysis and interpretation of results. MC participated in the statistical analysis and in the interpretation of results. JJG-R coordinated the acquisition of clinical data and participated in the analysis and interpretation of results. AG participated in the design of the study and in the coordination of acquisition of clinical data and collection of samples and supervised genotyping, statistical analysis, interpretation of results, and writing of the manuscript. All authors read and approved the final manuscript.

## Acknowledgements

We thank Carmen Pena-Pena for her excellent technical assistance and Yolanda Lopez-Golan for her help in recruiting patients. MS-G is the recipient of an FPU predoctoral bursary of the Spanish Ministry of Education. MC is the recipient of an 'Isabel Barreto' bursary of the government of Galicia. This project was supported by an unrestricted grant from Roche Spain and by grants PI080744 and PI09/90744 from the Instituto de Salud Carlos III (Spain) with participation of funds from FEDER (European Union).

## Supplementary Material

Additional file 1 **Primers and probes used for genotyping**. List of primers and probes used for genotyping the 16 SNPs included in the study. Click here for file

Additional file 2 **Details of some comparisons of response to treatment**. A table a table with the analyses done after 6 months of treatment and a table with the results of analyzing treatment response of patients receiving Infliximab at 3 months. Click here for file
