# Supplementary material for: Lack of replication of genetic predictors for the rheumatoid arthritis response to anti-TNF treatments: a prospective case-only study
Source: Arthritis Res Ther. 2010 Apr 27;12(2):R72. doi: 10.1186/ar2990 (PMC2888228; doi:10.1186/ar2990)
Supplement: Additional file 1 — Primers and probes used for genotyping. List of primers and probes used for genotyping the 16 SNPs included in the study. [file ar2990-S1.doc]

Primers and probes in 5’→ 3’ direction used for genotyping the 16 SNPs by the single-base extension approach.

|  | PCR primers | |  |
| --- | --- | --- | --- |
| SNP | Forward Primer | Reverse Primer | Minisequencing Probe |
| **rs983332** | atctgtagctctgtttctgcttgg | caagtactccttgctgccaaaag | cagactctctataattttaatggaatcaatacacaaggtccagcgtccaacta |
| **rs928655** | gcctttcccatcacattctc | ttctcagctgcctccttctt | CCAGAAGTATATTAATGAGCAGTGCgtgaattctgtgatttctcaccctgcctttcagatttg $ |
| **rs13393173** | tggtgttctcattgccttgaatg | gaacaagatggtctctatcccagg | tggaaagccctCagacagctacaatttagctaactcca |
| **rs437943** | ccagcattggtggaagtgat | tggtcagtttgaaaggtctgc | TTACCTATGATTGATgagcaccatatagaacttatagcaacca $ |
| **rs10945919** | tcactgcagttctgcctttg | tgatccactttgtgtttgga | tttggaaatttactattgaaagagaaaggttcaaagctgaaacattagctgcccaaggactca |
| **rs854547** | aagtggtcctcctgcttcag | ttggaccaagactagatttcctg | gctgcttttcaggagtgaatcactgaag |
| **rs854548** | aacaagccttgactgggtaaac | tgcaggctttgctcttttaac | TTACCTATGAtgactgggtaaacctgtatcaattgtcaaagGatatatctgtcaatca $ |
| **rs854555** | cccaagagatacgggctttaac | tttgtccacggtctttattcag | catcatatatatgatatcatcttgacatatttgtctactgctg |
| **rs868856** | cgctggtgcttcatactcac | agcctttgcagagacacctg | TTACCTATGAtcctagtccgtctttctctttcacttgtggttttcttcccttgtcatttaagcattctcaact $ |
| **rs7046653** | tgtaggcacaatgggtagctt | gagacactaggaccgtttgttg | tcctggtatcctttattttctgaattaaaaaaaaaaaaggctgtatga |
| **rs2814707** | aaacgacaagagaagcacagc | gcccacctcaaactaaagca | aattcctgcatccagtagtgcccaaagccagtg |
| **rs3849942** | ccatgctaggcactgagacac | cccactacagcccattcttc | ctttcttcccacaggtctagctagtacgtatttctttt |
| **rs774359** | agtaaaggaactaacatgtaggcactc | gtacccgtgggcaaggtg | cacctatttcctcatctgtaaaatggcaataatagtaatagtacctaatgtgt |
| **rs6138150** | caggatggaagctctgtcaag | agccaagactcagggtgtgt | gttggcaaattcagattccaaaagtctc |
| **rs6028945** | agctggaactgggtttgttg | gactcagccttgtggtgagg | tccaatgacagtagtgagtaaatgagcatgCactaaataaagaacaca |
| **rs6071980** | ctgattccttttcctgtttgc | tgcagaattcaaggacacaga | agcctgacaggatttttattttAtgtttggggaacagaatactaacaggtcatgttta |

Primers selected with Primer3 and Oligos softwares. They were checked to avoid formation of dimers between the primers included in the reaction.

$ These oligonucleotides were extended with a 5' tail that has not homology with human sequences (these tails are in capital letters)
