# Supplementary material for: Lack of replication of genetic predictors for the rheumatoid arthritis response to anti-TNF treatments: a prospective case-only study
Source: Arthritis Res Ther. 2010 Apr 27;12(2):R72. doi: 10.1186/ar2990 (PMC2888228; doi:10.1186/ar2990)
Supplement: Additional file 2 — Details of some comparisons of response to treatment. A table a table with the analyses done after 6 months of treatment and a table with the results of analyzing treatment response of patients receiving Infliximab at 3 months. [file ar2990-S2.doc]

**Table 1.** Analysis of the of the association between genotypes at the 16 SNPs and response to treatment with anti-TNF after 6 months of follow-up by linear regression with the relative change in DAS28 (relDAS28) and by comparison of responders and non-responders according to the EULAR criteria.

| SNP | *P* value of relDAS28 | Responders’ MAF % (n/N)^a^ | Non-responders’ MAF % (n/N)^a^ | O.R. (95% C.I.) | *P* value |
| --- | --- | --- | --- | --- | --- |
| rs983332 | 0.30 | 19.2 (23/120) | 26.7 (16/60) | 0.62 (0.3-1.4) | 0.23 |
| rs928655 | 0.23 | 19.4 (24/124) | 25.8 (16/62) | 0.68 (0.3-1.4) | 0.31 |
| rs13393173 | 0.45 | 17.7 (22/124) | 27.4 (17/62) | 0.61 (0.3-1.2) | 0.15 |
| rs437943 | 0.28 | 40.3 (50/124) | 30.6 (19/62) | 1.50 (0.8-2.9) | 0.21 |
| rs10945919 | 0.65 | 28.2 (35/124) | 29.0 (18/62) | 0.96 (0.5-1.9) | 0.91 |
| rs854547 | 0.89 | 35.5 (44/124) | 35.5 (22/62) | 1.00 (0.5-1.9) | 1.00 |
| rs854548 | 0.85 | 20.2 (25/124) | 21.0 (13/62) | 0.96 (0.5-1.9) | 0.91 |
| rs854555 | 0.61 | 33.9 (42/124) | 37.1 (23/62) | 0.87 (0.5-1.7) | 0.66 |
| rs868856 | 0.56 | 31.5 (39/124) | 33.9 (21/62) | 0.89 (0.5-1.7) | 0.74 |
| rs7046653 | 0.41 | 30.6 (38/124) | 33.9 (21/62) | 0.86 (0.4-1.7) | 0.65 |
| rs2814707 | 0.69 | 21.8 (27/124) | 24.2 (15/62) | 0.88 (0.4-1.8) | 0.72 |
| rs3849942 | 0.45 | 20.2 (25/124) | 24.2 (15/62) | 0.80 (0.4-1.6) | 0.54 |
| rs774359 | 0.53 | 24.2 (30/124) | 29.0 (18/62) | 0.78 (0.4-1.6) | 0.48 |
| rs6138150 | 0.054 | 16.1 (20/124) | 11.3 (7/62) | 1.43 (0.6-3.4) | 0.42 |
| rs6028945 | 0.75 | 12.1 (15/124) | 14.5 (9/62) | 0.78 (0.3-2.1) | 0.62 |
| rs6071980 | 0.84 | 18.5 (23/124) | 16.1 (10/62) | 1.22 (0.5-3.0) | 0.66 |

^a^ MAF = Minor Allele Frequency, n = number of minor alleles, N = total number of alleles

**Table 2.** Analysis of the association between genotypes at the 16 SNPs and response to treatment with Infliximab after 3 months of follow-up by linear regression with the relative change in DAS28 (relDAS28) and by comparison of responders and non-responders according to the EULAR criteria.

| SNP | *P* value of relDAS28 | Responders’ MAF % (n/N)^a^ | Non-responders’ MAF % (n/N)^a^ | O.R. (95% C.I.) | *P* value |
| --- | --- | --- | --- | --- | --- |
| rs983332 | 0.14 | 21.2 (14/66) | 27.3 (12/44) | 0.72 (0.3-1.7) | 0.46 |
| rs928655 | 0.85 | 20.6 (14/68) | 18.2 (8/44) | 1.17 (0.4-3.1) | 0.75 |
| rs13393173 | 0.86 | 22.1 (15/68) | 25.0 (11/44) | 0.85 (0.3-2.1) | 0.72 |
| rs437943 | 0.67 | 36.8 (25/68) | 25.0 (11/44) | 1.74 (0.8-4.0) | 0.19 |
| rs10945919 | 0.47 | 27.9 (19/68) | 22.7 (10/44) | 1.32 (0.5-3.2) | 0.54 |
| rs854547 | 0.70 | 35.3 (24/68) | 36.4 (16/44) | 0.95 (0.45-2.1) | 0.91 |
| rs854548 | 0.81 | 22.1 (15/68) | 25.0 (11/44) | 0.85 (0.3-2.1) | 0.72 |
| rs854555 | 0.92 | 30.9 (21/68) | 36.4 (16/44) | 0.78 (0.4-1.7) | 0.55 |
| rs868856 | 0.92 | 41.2 (28/68) | 40.9 (18/44) | 1.01 (0.5-2.2) | 0.98 |
| rs7046653 | 0.71 | 39.7 (27/68) | 40.9 (18/44) | 0.95 (0.4-2.1) | 0.9 |
| rs2814707 | 0.83 | 30.9 (21/68) | 31.8 (14/44) | 0.96 (0.4-2.2) | 0.92 |
| rs3849942 | 0.48 | 27.9 (19/68) | 31.8 (14/44) | 0.83 (0.4-1.9) | 0.66 |
| rs774359 | 0.17 | 30.9 (21/68) | 43.2 (19/44) | 0.59 (0.3-1.3) | 0.18 |
| rs6138150 | 0.78 | 13.2 (9/68) | 18.2 (8/44) | 0.69 (0.2-1.9) | 0.48 |
| rs6028945 | 0.39 | 13.2 (9/68) | 9.1 (4/44) | 1.53 (0.4-5.3) | 0.5 |
| rs6071980 | 0.48 | 22.1 (15/68) | 13.6 (6/44) | 1.79 (0.6-5.0) | 0.26 |

^a^ MAF = Minor Allele Frequency, n = number of minor alleles, N = total number of alleles
